# *Porphyromonas endodontalis* HmuY differentially participates in heme acquisition compared to the *Porphyromonas gingivalis* and *Tannerella forsythia* hemophore-like proteins

**DOI:** 10.3389/fcimb.2024.1421018

**Published:** 2024-06-13

**Authors:** Michał Śmiga, Teresa Olczak

**Affiliations:** Laboratory of Medical Biology, Faculty of Biotechnology, University of Wrocław, Wrocław, Poland

**Keywords:** *Porphyromonas endodontalis*, *Porphyromonas gingivalis*, *Tannerella forsythia*, hemophore-like protein, HmuY, heme

## Abstract

**Introduction:**

*Porphyromonas gingivalis* and *Porphyromonas endodontalis* belong to the Bacteroidota phylum. Both species inhabit the oral cavity and can be associated with periodontal diseases. To survive, they must uptake heme from the host as an iron and protoporphyrin IX source. Among the best-characterized heme acquisition systems identified in members of the Bacteroidota phylum is the *P. gingivalis* Hmu system, with a leading role played by the hemophore-like HmuY (HmuY^Pg^) protein.

**Methods:**

Theoretical analysis of selected HmuY proteins and spectrophotometric methods were employed to determine the heme-binding mode of the *P. endodontalis* HmuY homolog (HmuY^Pe^) and its ability to sequester heme. Growth phenotype and gene expression analysis of *P. endodontalis* were employed to reveal the importance of the HmuY^Pe^ and Hmu system for this bacterium.

**Results:**

Unlike in *P. gingivalis*, where HmuY^Pg^ uses two histidines for heme-iron coordination, other known HmuY homologs use two methionines in this process. *P. endodontalis* HmuY^Pe^ is the first characterized representative of the HmuY family that binds heme using a histidine-methionine pair. It allows HmuY^Pe^ to sequester heme directly from serum albumin and *Tannerella forsythia* HmuY^Tf^, the HmuY homolog which uses two methionines for heme-iron coordination. In contrast to HmuY^Pg^, which sequesters heme directly from methemoglobin, HmuY^Pe^ may bind heme only after the proteolytic digestion of hemoglobin.

**Conclusions:**

We hypothesize that differences in components of the Hmu system and structure-based properties of HmuY proteins may evolved allowing different adaptations of *Porphyromonas* species to the changing host environment. This may add to the superior virulence potential of *P. gingivalis* over other members of the Bacteroidota phylum.

## 1 Introduction

Bacteria belonging to the *Porphyromonas* genus (Gibson and Genco, 2006; Summanen et al., 2015; Guilloux et al., 2021) inhabit mainly the oral cavity, gastrointestinal tract, and urogenital tract of humans, domestic and wild animals (Paster et al., 1994; Finegold et al., 2004; Sakamoto and Ohkuma, 2013; Zamora-Cintas et al., 2018; Acuna-Amador and Barloy-Hubler, 2020; Guilloux et al., 2021; Morales-Olavarria et al., 2023). Usually, they are isolated from oral infections (e.g., gingivitis, periodontitis, endodontic infections, abscesses) but also from other body infections (e.g., abscesses, infections of amniotic fluid and umbilical cord, infected wound sites) (van Steenbergen et al., 1984; Sundqvist, 1992; Zamora-Cintas et al., 2018; Acuna-Amador and Barloy-Hubler, 2020; Morales-Olavarria et al., 2023). Some of them, like *Porphyromonas gingivalis*, belong to opportunistic pathogens found in healthy oral cavities in low numbers but in high numbers in patients with periodontitis (Ximenez-Fyvie et al., 2000; Gomes et al., 2005; Bik et al., 2010).

*Porphyromonas endodontalis* is a Gram-negative, anaerobic, asaccharolytic, black-pigmented bacterium, primarily associated with root canal infections, often identified in dental periapical abscesses of endodontic origin and orofacial odontogenic infections (Sundqvist et al., 1979; van Steenbergen et al., 1984; van Winkelhoff et al., 1985a; Sundqvist et al., 1989; Sundqvist, 1992; Gomes et al., 2005; Siqueira and Rocas, 2009; Flynn et al., 2012). The most prevalent species in persistent endodontic infections, aside from *P. endodontalis*, comprise other members of the Bacteroidota (formerly Bacteroidetes) phylum, mainly *P. gingivalis*, *Tannerella forsythia*, and *Prevotella intermedia* (Gomes et al., 2005; Siqueira and Rocas, 2009; Flynn et al., 2012; Pinto et al., 2023). *P. endodontalis* can also be found together with *P. gingivalis*, *T. forsythia*, and *P. intermedia* in diseased periodontal sites in patients with periodontitis, mainly in apical periodontitis originating from endodontic infection (Tran et al., 1997; Kumar et al., 2003; Pereira et al., 2011; Lombardo Bedran et al., 2012; Lourenco et al., 2014; Perez-Chaparro et al., 2014; Na et al., 2020; Jimenez et al., 2022; Alvarez et al., 2023). Among *Porphyromonas* species, the best characterized *P. gingivalis* is not only the main etiologic agent and keystone pathogen of periodontitis (Darveau et al., 2012; Hajishengallis and Lamont, 2012; Deng et al., 2017, 2018; Hajishengallis and Diaz, 2020) but is often associated with systemic inflammation-based diseases (Nazir, 2017; Mei et al., 2020).

Although *P. endodontalis* and *P. gingivalis* belong to the same genus, both species differ in their phenotypes. In contrast to *P. gingivalis*, *P. endodontalis* is more sensitive to oxygen, grows better in culture media supplemented with heme, hemoglobin, or PPIX, does not exhibit hemagglutination activity, does not produce trypsin-like proteolytic enzymes and gingipains (van Steenbergen et al., 1984; van Winkelhoff et al., 1985b, 1986; Zerr et al., 2000, 2001). Similar to *P. gingivalis*, it produces collagenases and other proteases, such as dipeptidyl-peptidases (Sorsa et al., 1992; Tran et al., 1997; Odell et al., 1999; Chang et al., 2002; Nishimata et al., 2014), and can degrade host proteins, including hemoglobin (Kilian, 1981; Carlsson et al., 1984; Jansen et al., 1994; Rosen et al., 2001). Due to the lack of genes encoding a functional heme biosynthesis pathway in the *P. endodontalis* genome (GenBank accession number: ACNN00000000.1), typical for almost all members of the Bacteroidota phylum, *P. endodontalis* must uptake this compound from the host as an iron and protoporphyrin IX (PPIX) source.

Among the best-characterized heme acquisition systems identified in members of the Bacteroidota phylum is the *P. gingivalis* Hmu system (Wojtowicz et al., 2009a, Wojtowicz et al., 2009b; Bielecki et al., 2018, 2020; Sieminska et al., 2021; Antonyuk et al., 2023). The Hmu system comprises six proteins (HmuYRSTUV) encoded on the *P. gingivalis hmu* operon. The first protein encoded on this operon, HmuY^Pg^, is the first representative of the novel HmuY family comprising hemophore-like proteins, different from classical hemophores or other hemophore-like proteins, such as *P. gingivalis* HusA protein (Olczak et al., 2024). Although *P. gingivalis* can transport free heme directly using TonB-dependent outer-membrane receptor HmuR^Pg^, encoded downstream of the *hmuY^Pg^* gene, HmuY^Pg^ protein facilitates this process through binding of heme and its delivery to HmuR^Pg^ (Smalley and Olczak, 2017). Importantly, HmuY^Pg^ can sequester heme directly from host hemoproteins or heme-binding proteins produced by cohabitating bacteria and deliver it to HmuR^Pg^ (Smalley and Olczak, 2017; Olczak et al., 2024). The functions of the other proteins encoded on the *P. gingivalis hmu* operon are unknown and they most likely play a role in heme transport into the bacterial cell and/or heme metabolism (Smalley and Olczak, 2017; Rocha et al., 2019).

Our research has shown that proteins belonging to the HmuY family differ in their heme-binding properties (Olczak et al., 2024), which may influence the adaptation of bacteria to the host environment they occupy. The most significant difference among HmuY homologs is the type of amino acids engaged in heme-iron coordination, which results in different heme-binding capacities depending on the heme-iron redox state. So far characterized HmuY proteins coordinating heme iron by two methionines (HmuY homologs from *P. intermedia*, *T. forsythia*, *Bacteroides vulgatus*, and *Bacteroides fragilis*) bind heme preferentially under reducing conditions (Bielecki et al., 2018, 2020; Sieminska et al., 2021; Antonyuk et al., 2023), while *P. gingivalis* HmuY coordinates heme iron by two histidines which results in a high affinity of heme binding in both oxidized and reduced environments (Wojtowicz et al., 2009a, Wojtowicz et al., 2009b).

In this study, we characterized another member of the HmuY family, a hemophore-like protein produced by *P. endodontalis* (HmuY^Pe^), and compared its properties with *P. gingivalis* HmuY^Pg^ and *T. forsythia* HmuY^Tf^. This approach allowed us to understand better the function and properties of HmuY family proteins produced by different human pathogens.

## 2 Materials and methods

### 2.1 Bacterial strains and growth conditions

*P. endodontalis* ATCC 35406 (Argenta, Poznań, Poland) and *P. gingivalis* A7436 (laboratory collection) strains were grown on Schaedler blood agar (ABA) plates (Argenta) at 37°C for 6 and 4 days, respectively, under anaerobic conditions (80% N_2_, 10% H_2_ and 10% CO_2_) (Whitley A35 anaerobic workstation; Bingley, UK). Then, bacteria were used to inoculate a liquid basal medium (BM) composed of 3% trypticase soy broth (Becton Dickinson, Sparks, MD, USA) and 0.5% yeast extract (Biomaxima, Lublin, Poland), supplemented with 0.05% L-cysteine (Carl Roth, Karlsruhe, Germany), 0.5 mg/l menadione (Sigma-Aldrich, St. Louis, MO, USA) (BM medium), and 7.7 µM hemin chloride (Fluka, Munich, Germany) to allow bacteria to grow in optimal, iron- and heme-rich conditions (Hm medium). Alternatively, to starve bacteria of iron and heme, no heme source was added and the medium was supplemented with 160 µM 2,2-dipyridyl (Sigma-Aldrich) to complex free iron (DIP medium). The optical density at 600 nm (OD_600_) at the beginning of the liquid culture was at least 0.5 and 0.2 for *P. endodontalis* and *P. gingivalis*, respectively.

To analyze growth curves, bacteria were cultured for two passages in BM medium without the addition of a heme source. BM medium (BM alone) or medium supplemented with 1.25 or 5 µM human hemoglobin (Hb), BM medium supplemented with 5 µM heme and 5 µM human serum albumin (HSA), 5 µM HmuY^Pg^, 5 µM HmuY^Pe^, or 5 µM HmuY^Tf^ were inoculated with *P. gingivalis* or *P. endodontalis* at starting OD_600_ equal to 0.2 or 0.5, respectively. Media supplemented with proteins and heme were preincubated for 16 hours at 4°C before use, to allow saturation of proteins with heme.

*Escherichia coli* ER2566 strain (New England Biolabs, Ipswich, MA, USA) was grown under standard aerobic conditions.

### 2.2 Plasmid construction, mutagenesis, protein overexpression, and protein purification

The recombinant HmuY^Pe^, lacking the predicted signal peptide (MKTRFFLALIATSLVLGVASCRP), was overexpressed and purified using affinity chromatography. Briefly, the *hmuY^Pe^* gene from *P. endodontalis* (GenBank accession number: *POREN0001_0444*) was PCR amplified using primers listed in **Supplementary Table S1** and cloned into XcmI and BamHI restriction sites of a pTriEx-4 plasmid (Sigma-Aldrich) using NEBuilder HiFi DNA Assembly (New England Biolabs), resulting in the plasmid encoding HmuY^Pe^ protein with an N-terminal 6×His tag and the site recognized by Factor Xa.

To generate plasmids encoding HmuY^Pe^ variants with amino acid substitutions, the QuikChange II XL Site-Directed Mutagenesis Kit (Agilent Technologies, Santa Clara, CA, USA) and primers listed in **Supplementary Table S1** were used.

HmuY^Pe^ protein and its site-directed mutagenesis variants were overexpressed in *E. coli* after induction with 0.5 mM isopropyl-ß-D-thiogalacto-pyranoside (IPTG; Carl-Roth) at 16°C for 16 hours. Proteins were purified using the soluble fraction of *E. coli* cell lysates and TALON Superflow resin according to the manufacturer’s instructions (Sigma-Aldrich), using 25 mM HEPES buffer, pH 7.8, supplemented with 300 mM NaCl. For the elution step, 25 mM Tris/HCl buffer, pH 7.6, supplemented with 80 mM NaCl and 150 mM imidazole (Carl-Roth) was used. To obtain un-tagged proteins, the buffer was exchanged for 25 mM Tris/HCl, pH 7.6, supplemented with 80 mM NaCl. The 6×His tag was removed using Factor Xa (New England Biolabs) and filtration through an Amicon Ultra-4 Centrifugal Ultracel-10KDa filter unit (Millipore). If necessary, another step of affinity chromatography with nickel-immobilized resin (Ni-NTA; New England Biolabs) was used to remove the 6×His tag before protein concentration (**Supplementary Figure S2**).

HmuY^Pg^ protein from *P. gingivalis* and HmuY^Tf^ protein from *T. forsythia* were overexpressed and purified as described previously (Slezak et al., 2020; Śmiga et al., 2023a). To determine protein concentration, empirical molar absorption coefficients for HmuY^Pg^ (ϵ_280_ = 36.86 mM^−1^ cm^−1^) (Wojtowicz et al., 2009b) and HmuY^Tf^ (ϵ_280_ = 26.32 mM^−1^ cm^−1^) (Bielecki et al., 2018) were used. For *P. endodontalis* HmuY^Pe^, the empirical molar absorption coefficient was determined in this study (ϵ_280_ = 35.56 mM^−1^ cm^−1^), as described by others (Eakanunkul et al., 2005).

### 2.3 Sodium dodecyl sulfate-polyacrylamide gel electrophoresis, Western blotting, and dot blotting

Protein samples, *P. endodontalis* and *P. gingivalis* whole cell lysates, or samples prepared from the whole bacterial cultures were analyzed by SDS-PAGE. Samples were separated on 12% polyacrylamide gels and the proteins were visualized with Coomassie Brilliant Blue G-250 (CBB G-250) or were transferred onto nitrocellulose membranes (Millipore, Billerica, MA, USA). 20 or 100 ng of HmuY^Pe^, HmuY^Pg^, or HmuY^Tf^ proteins in 5 µl were applied onto nitrocellulose membranes for dot blotting. Western blotting and dot blotting were performed as described before (Śmiga et al., 2015; Śmiga et al., 2023a). Briefly, membranes were incubated with rabbit anti-HmuY^Pg^ or rabbit anti-HmuY^Tf^ polyclonal antibodies (1:10,000; GenScript USA Inc.). Subsequently, goat anti-rabbit IgG antibodies conjugated with horseradish peroxidase (1:10,000; Sigma-Aldrich) were applied. Chemiluminescence staining (Perkin Elmer, Waltham, MA, USA) and ChemiDoc Imaging System (Bio-Rad Laboratories, Hercules, CA, USA) were used to visualize proteins.

### 2.4 Heme-protein complex formation

Analysis of heme binding was performed as described previously (Śmiga et al., 2023b). Briefly, ~8 mg of hemin chloride (Pol-Aura, Morąg, Poland) was dissolved in 0.1 M NaOH, and its concentration was determined using empirical molar absorption coefficient (ϵ_385_ = 58.5 mM^−1^ cm^−1^). Proteins at 5 µM concentration were prepared in 20 mM sodium phosphate buffer, pH 7.4, containing 140 mM NaCl (PBS). Protein samples were titrated with heme, and protein-heme complexes were monitored by UV-visible absorbance (250–700 nm) spectroscopy with a double-beam Jasco V-750 spectrophotometer (Jasco GmbH, Pfungstadt, Germany). The reduced conditions were formed by the addition of sodium dithionite (Sigma-Aldrich) to the final 10 mM concentration and mineral oil overlay of the sample (Sigma-Aldrich).

### 2.5 Heme sequestration experiments

Heme sequestration was examined by mixing holo- (protein-heme complex) and apo-protein (protein alone) in PBS and monitoring heme transfer by UV-visible absorbance spectroscopy or PAGE, as described before (Smalley et al., 2011; Śmiga et al., 2023b). Except for methemoglobin (metHb; Sigma-Aldrich), protein-heme complexes were prepared by mixing protein and heme at a 1:1.2 molar ratio and incubation at room temperature for 1 hour. To remove unbound heme, the solutions were passed through Zeba Spin desalting columns (Thermo Fisher, Scientific, Waltham, MA, USA).

1.25 µM metHb or 5 µM other holo-proteins were mixed with 5 µM apo-proteins. The heme sequestration process was monitored over time by recording UV-visible absorbance spectra under oxidizing and reducing conditions. Alternatively, 20 µM holo-HmuY^Tf^ protein or 10 µM other holo-proteins were mixed with 10 µM apo-proteins and incubated for 30 minutes at 37°C. To 30 µl of the sample, 10 µl of 0.4 M Tris/HCl buffer, pH 6.8, supplemented with 40% glycerol and 0.08% bromophenol blue was added, and 25 µl of the sample was immediately loaded on the 13.5% PAGE-separating gel prepared without SDS. After electrophoresis, the heme-containing complexes were first stained using 5 mM 3,3′,5,5′-tetramethylbenzidine (TMB) prepared in 100 mM Tris/HCl buffer, pH 7.5, supplemented with 140 mM NaCl and 0.05% H_2_O_2_ (TMB-H_2_O_2_ staining) up to 30 minutes. Subsequently, all proteins were stained with CBB G-250.

### 2.6 Analysis of gene expression using reverse transcriptase-quantitative polymerase chain reaction

To determine the influence of iron and heme on *P. endodontalis* gene expression, bacteria were cultured in Hm medium or DIP medium for one or two 24-hour passages. Bacteria from 1 ml of culture were centrifuged and used for RNA isolation with the Total RNA Mini Kit (A&A Biotechnology, Gdańsk, Poland). Genomic DNA contamination was removed using the Clean-up RNA concentrator Kit (A&A Biotechnology). RNA was used to generate cDNA with a LunaScript RT SuperMix Kit (New England Biolabs). qPCR was performed using SensiFAST SYBR no-ROX Kit (Bioline, London, UK) and LightCycler 96 (Roche, Basel, Switzerland). PCR program comprised initial denaturation at 95°C for 120 seconds, 35 cycles of denaturation at 95°C for 5 seconds, primers annealing at 60°C for 10 seconds, and extension at 72°C for 15 seconds. After PCR, the melting curves were generated for PCR quality control. Relative change in gene expression was calculated using LightCycler 96 software (Roche) and *16S rRNA* as a reference gene. All analyses were carried out in 4 biological repetitions. All primers used are listed in **Supplementary Table S1**.

### 2.7 Determination of proteolytic activity

The total proteolytic activity of whole bacterial cultures was measured using azocasein (Sigma-Aldrich) as a substrate. Briefly, to 40 μl of 1.5% azocasein solution in reaction buffer (20 mM Tris/HCl buffer, pH 7.5, supplemented with 150 mM NaCl, 5 mM CaCl_2_, 0.05% Tween 20, and 10 mM L-cysteine hydrochloride freshly neutralized with NaOH), 10 μl of total *P. endodontalis* or *P. gingivalis* overnight culture (grown for 24 hours) was added. Samples were incubated for 30 minutes at 37°C, and the reaction was stopped by adding 200 μl of 5% TCA (Sigma-Aldrich). Precipitated, undigested azocasein was separated by centrifugation (2000×*g*, 10 min). 100 μl of the supernatant was mixed with 60 μl of 0.5 M NaOH and the absorbance at 450 nm (A_450_) was measured using a GloMax Discover plate reader (Promega, Madison, WI, USA). The final results were presented as a change of A_450_ over 60 minutes caused by 1 ml of culture exhibiting OD_600_ equal to 1 (ΔA_450_/60 min/ml).

### 2.8 The susceptibility of proteins to proteolysis

HSA (Sigma-Aldrich), metHb (Sigma-Aldrich), hemopexin (Hpx; Sigma-Aldrich), *P. gingivalis* HmuY^Pg^, *P. endodontalis* HmuY^Pe^, and *T. forsythia* HmuY^Tf^ were used to analyze their susceptibility to proteolysis performed by proteases produced by *P. endodontalis*. Briefly, Hm medium was supplemented with 2 µM proteins and the bacterial cultures were started with the initial OD_600_ equal to 0.5. Samples were collected over time and examined using SDS-PAGE and staining with CBB-G250. As a control, the fresh BM+Hm medium or *P. gingivalis* culture started at OD_600_ equal to 0.2 was used.

### 2.9 Statistical analyses

All experiments were performed independently at least in two biological replicates and at least in three technical repetitions each. The numerical values are represented as mean ± standard deviation (mean ± SD) or mean ± standard error (mean ± SE). All statistical analyses were done using unpaired Student’s *t*-test with GraphPad software (GraphPad Prism 8.0 Inc., San Diego, CA, USA).

### 2.10 Bioinformatics analyses

Search for protein sequences was performed in the GenBank database using PSI-BLAST (https://blast.ncbi.nlm.nih.gov/Blast.cgi). Protein amino acid sequences were compared with The Clustal Omega (Madeira et al., 2022) and Jalview (Waterhouse et al., 2009). Protein similarity and identity were analyzed using Sequence Manipulation Suite: Ident and Sim (Stothard, 2000). Solved (RCSB Protein Data Bank; https://www.rcsb.pdb) and predicted with AlphaFold Protein Structure Database (Jumper et al., 2021; Varadi et al., 2022) three-dimensional protein structures were visualized with UCSF Chimera (Pettersen et al., 2004). Heme binding in HmuY^Pe^ was predicted with EDock (Zhang et al., 2020). Prediction of *P. endodontalis hmu* operon genes was performed using an Operon mapper (https://biocomputo.ibt.unam.mx/operon_mapper/).

## 3 Results

### 3.1 Characterization of Hmu system in *Porphyromonas endodontalis*

**Figure 1:**
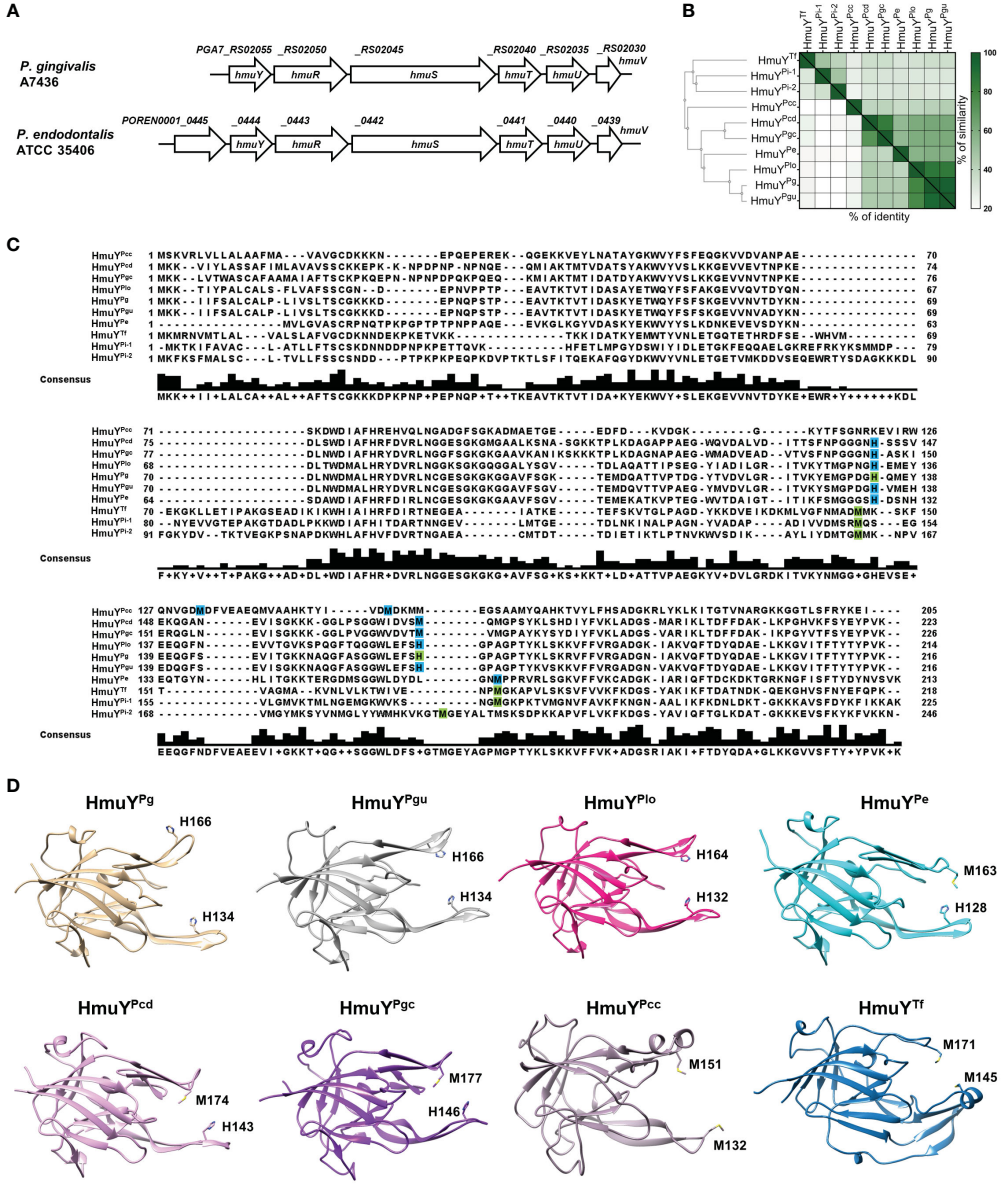
Theoretical analysis of the *P. endodontalis* Hmu system. **(A)** Organization of *P. endodontalis hmu* operon and its counterpart in *P. gingivalis*. **(B)** Homology of the HmuY^Pe^ protein to the representatives of the HmuY protein family presented in the form of a heat map indicating the % identity and similarity between the amino acid sequences of the proteins. The simplified guide tree was created with Clustal Omega (https://www.ebi.ac.uk/jdispatcher/msa/clustalo). **(C)** The alignment of HmuY protein sequences with marked residues involved in heme-iron coordination experimentally confirmed (green) or theoretically predicted (blue). The consensus amino acid sequence is shown below the examined sequences. HmuY homologs from *P. gingivalis* (HmuY^Pg^), *P. gulae* (HmuY^Pgu^), *P. endodontalis* (HmuY^Pe^), *P. loveana* (HmuY^Plo^), *P. gingivicanis* (HmuY^Pgc^), *P. circumdentaria* (HmuY^Pcd^), *P. crevioricanis* (HmuY^Pcc^), *T. forsythia* (HmuY^Tf^) and *P. intermedia* (HmuY^Pi-1^ and HmuY^Pi-2^). **(D)** Comparison of overall structures of representative HmuY proteins produced by *Porphyromonas* species and *T. forsythia*, with marked heme-iron coordinating amino acids. Structures of Hmu^Pg^ and HmuY^Tf^ determined by crystallography have been deposited under PDB IDs: 6EWM and 6EU8, respectively. Other structures of HmuY homologs were predicted with AlphaFold: HmuY^Pgu^ (ID: AF-A0A099WTH2-F1), HmuY^Pe^ (ID: AF-C3JC46-F1), HmuY^Plo^ (ID: AF-A0A2U1FHF1-F1), HmuY^Pgc^ (ID: AF-A0A0A2GAC0-F1), HmuY^Pcd^ (ID: AF-A0A1T4NY32-F1), and HmuY^Pcc^ (ID: AF-A0A0A2FIS2-F1).

*P. endodontalis* ATCC 35406 *hmu* operon organization is similar to that found in *P. gingivalis*, including one HmuY homolog (**Figure 1A**). HmuY^Pe^ protein is closely related to HmuY^Pg^ with ~50% amino acid sequence identity, as well as 21–22% identity and 32% similarity to other characterized HmuY proteins produced by human oral pathogens from the Bacteroidota phylum (**Figures 1B, C**). Other proteins of the Hmu system in *P. endodontalis* show high homology to those found in *P. gingivalis* with amino acid sequence identity as follows: HmuR ~49%, HmuS ~ 65%, HmuT ~50%, HmuU ~68%, and HmuV ~79%. Interestingly, in *P. endodontalis* an additional gene (GenBank accession number: *POREN0001_0445*) is located upstream of the *hmuY^Pe^* gene, encoding a protein containing T9SS type A sorting domain (T9SS) (**Figure 1A**). Using theoretical and experimental approaches, we confirmed that the *POREN0001_0445* gene is a part of the *hmu* operon (**Supplementary Figure S1A**). Proteins homologous to the protein encoded by the *POREN0001_0445* gene are found in other bacteria including some *Porphyromonas* species and *P. intermedia*. However, in contrast to *P. endodontalis*, they are not encoded as a part of operons but as orphan genes (data not shown). Despite the low amino acid sequence identity between analyzed homologs of the POREN0001_0445 protein (**Supplementary Figure S1B**), their predicted tertiary structure is highly similar (**Supplementary Figure S1C**). They form a β-barrel-like structure, which may suggest their role in transporting an unknown molecule through the outer membrane.

### 3.2 *P. endodontalis* HmuY^Pe^ binds heme

Analysis of the overall three-dimensional experimentally solved (*P. gingivalis* HmuY^Pg^ and *T. forsythia* HmuY^Tf^) or theoretically predicted (other selected *Porphyromonas* species) protein structures revealed that HmuY proteins identified in the *Porphyromonas* species are highly similar to the *P. gingivalis* HmuY^Pg^ (**Figure 1D**), with the highest similarities observed in the core region (Śmiga et al., 2023a). As in so far characterized HmuY proteins (Olczak et al., 2024), the main differences in HmuY proteins identified in *Porphyromonas* species are visible in the structure of heme-binding pockets and differ mainly in the size of the entrance of the heme-binding pocket (**Figure 1D**). The data shown in **Figure 1** allowed us to verify the spatial location of predicted amino acids involved in heme binding in HmuY homologs in analyzed *Porphyromonas* species. To date, the characterization of HmuY proteins has included the HmuY^Pg^ protein from the *P. gingivalis*, which uses two histidines for heme-iron coordination, and HmuY homologs which use two methionines in this process (for example *T. forsythia* HmuY^Tf^, *P. intermedia* HmuY^Pi-1^ and HmuY^Pi-2^). Our theoretical analyses showed that the majority of HmuY proteins identified in *Porphyromonas* species most likely coordinate heme-iron using a histidine-methionine pair or two methionines (**Figures 1C, D**). Only proteins closely related to the HmuY^Pg^, namely HmuY^Pgu^ from *P. gulae* and HmuY^Plo^ from *P. loveana* may coordinate heme iron with two histidines (**Figures 1C, D**).

**Figure 2:**
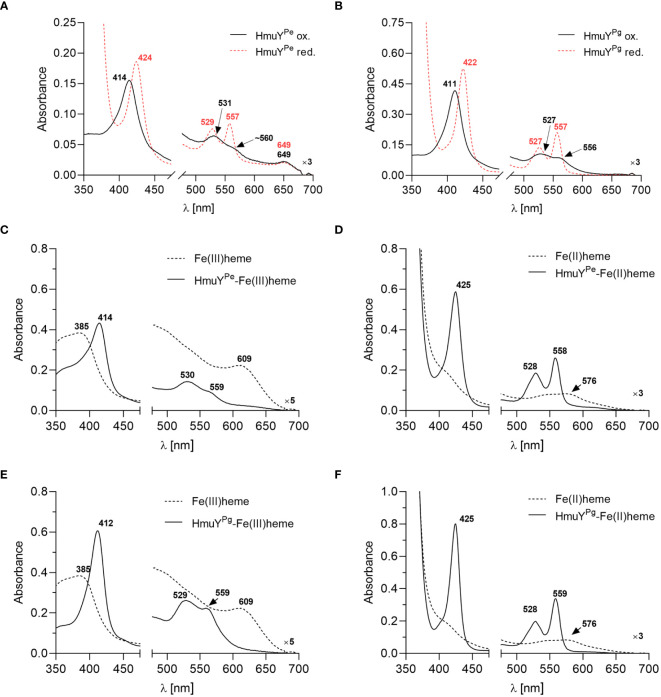
The heme-binding ability of HmuY proteins. The UV-visible absorbance spectrum of 50 µM purified *P. endodontalis* HmuY^Pe^ protein indicates heme binding **(A)**, similar to the 50 µM purified *P. gingivalis* HmuY^Pg^ **(B)**, examined under oxidizing (black lines) and reducing conditions (red lines), the latter formed by 10 mM sodium dithionite. Heme binding by HmuY^Pe^ **(C, D)** or HmuY^Pg^ **(E, F)** was confirmed by saturating the protein with heme at a 1:1.2 molar ratio and removing the excess heme by desalting. The UV-visible absorbance spectrum of the obtained sample (5 µM protein with bound heme) was compared to the spectrum of the 5 µM heme alone sample under oxidizing **(C, E)** and reducing conditions **(D, F)**.

To confirm the heme binding ability of the HmuY^Pe^, the protein was overexpressed in *E. coli* and purified using chromatographic methods (**Supplementary Figures S2A**, **S2B**). Purified and concentrated protein samples exhibited a reddish color (data not shown) similar to the HmuY^Pg^ sample (Bielecki et al., 2018, 2020). The UV-visible spectroscopic analysis demonstrated that under both oxidizing and reducing conditions, the HmuY^Pe^ sample exhibited spectra similar to those of the purified HmuY^Pg^ sample (**Figures 2A, B**), which indicated heme binding. To confirm this, the HmuY^Pe^ protein was saturated with heme [Fe(III)heme], and heme excess was removed by desalting. A UV-Vis absorbance spectrum of the sample was characterized by maxima in both the Soret band (414 nm) and Q bands (530 nm and 559 nm) (**Figure 2C**). Reduction of heme in this sample [Fe(II)heme] resulted in a red shift in the Soret band (425 nm), and a shift of the Q bands to 528 nm and 558 nm, which became more intense and resolved (**Figure 2D**). These spectra were similar to those obtained for the HmuY^Pg^-heme complex (**Figures 2E, F**).

**Figure 3:**
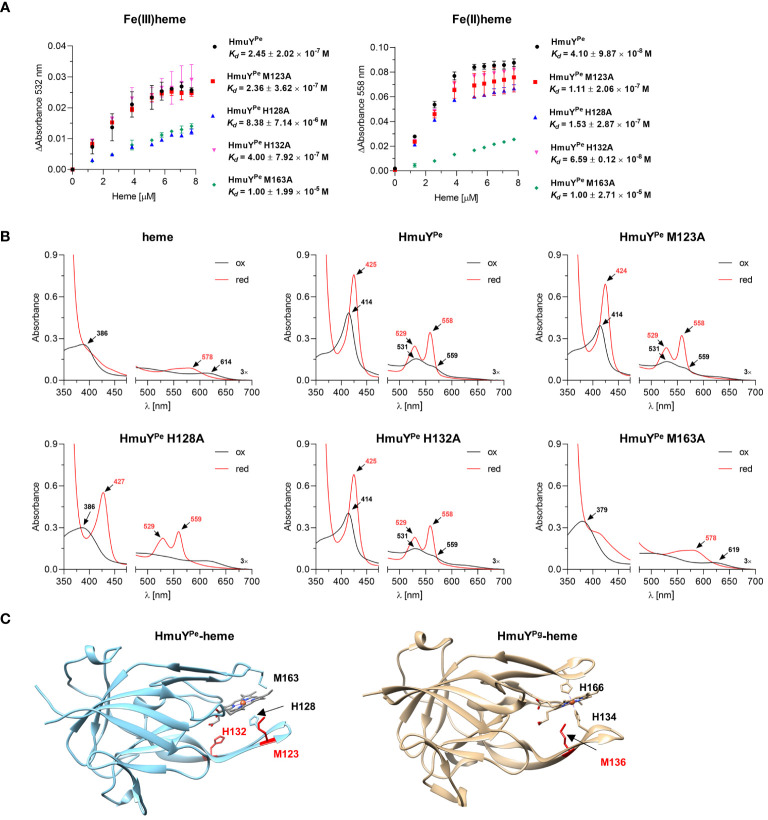
Analysis of *P. endodontalis* HmuY^Pe^ protein site-directed mutagenesis variants in complex with heme. **(A)** To determine the dissociation constant (*K_d_*), HmuY^Pe^ protein variants were titrated with increasing heme concentrations. Difference absorbance spectra were used for plotting graphs of the change in the Q band absorbance maximum for oxidizing (ΔAbsorbance at 532 nm) and reducing (ΔAbsorbance at 558 nm) conditions *versus* heme concentration. *K_d_* was determined using the one-site binding model. **(B)** Purified HmuY^Pe^ and its variants with single substitutions of methionine or histidine for alanine were saturated with heme at a 1:1 molar ratio. UV-visible absorbance spectra were recorded under oxidizing (black lines) and reducing (red lines) conditions, and compared to the spectrum of heme alone and the unmodified HmuY^Pe^ protein. The reducing conditions were formed by 10 mM sodium dithionite. **(C)** Comparison of overall protein structures of HmuY^Pe^ and HmuY^Pg^ in complex with heme. The experimentally solved three-dimensional structure of *P. gingivalis* HmuY^Pg^ in complex with heme (PDB ID: 3H8T) and modeled predicted structure of *P. endodontalis* HmuY^Pe^ in complex with heme are shown. The heme-iron coordinating amino acids are indicated in black, and amino acids with supporting or putatively supporting roles in heme binding are marked in red.

Heme binding strength was analyzed by determination of the HmuY^Pe^-heme complex dissociation constant (*K_d_*) (**Figure 3A**) by titration of apo-protein with increasing concentrations of heme (**Supplementary Figure S3**). *K_d_* of HmuY^Pe^-heme complex was lower under reducing (4.10 × 10^-8^ M) than oxidizing (2.45 × 10^-7^ M) (**Figure 3A**) conditions, thus being similar to HmuY homologs coordinating heme-iron with two methionine residues, e.g., *T. forsythia* HmuY^Tf^, than to *P. gingivalis* HmuY^Pg^ (Bielecki et al., 2018).

To experimentally confirm heme-iron coordinating amino acids, we prepared HmuY^Pe^ site-directed mutagenesis protein variants with selected histidines or methionines replaced by an alanine (M123A, H128A, H132A, and M163A), chosen based on the comparative analysis of amino acid sequences (**Figure 1C**). Particular single mutagenesis variants were overexpressed in *E. coli* and purified at similar levels as the unmodified protein (**Supplementary Figure S2C**). All protein variants were stable after purification, which suggests that amino acid replacements did not influence the tertiary protein structure. UV-visible absorbance spectra of HmuY^Pe^-heme complexes showed changes mostly in the case of H128A and M163A protein variants, suggesting that these amino acids could be engaged in heme-iron coordination (**Figure 3B**). The determination of *K_d_* showed that the H128A and M163A variants are characterized by a lower affinity for heme than an unmodified protein (**Figure 3A**). A slightly lower affinity of heme binding under reducing conditions in the case of the M123A variant (**Figure 3A**) may suggest local structural changes in the loop engaged in heme-iron coordination and/or supportive role in heme binding as it was shown for HmuY^Pg^ M136 (Wojtowicz et al., 2009b; Bielecki et al., 2018). This finding was confirmed by our theoretical analysis using the modeled HmuY^Pe^ structure and its comparison to the experimentally solved HmuY^Pg^-heme structure (**Figure 3C**).

### 3.3 Histidines are an evolutionarily gained advantage in heme acquisition by HmuY proteins

**Figure 4:**
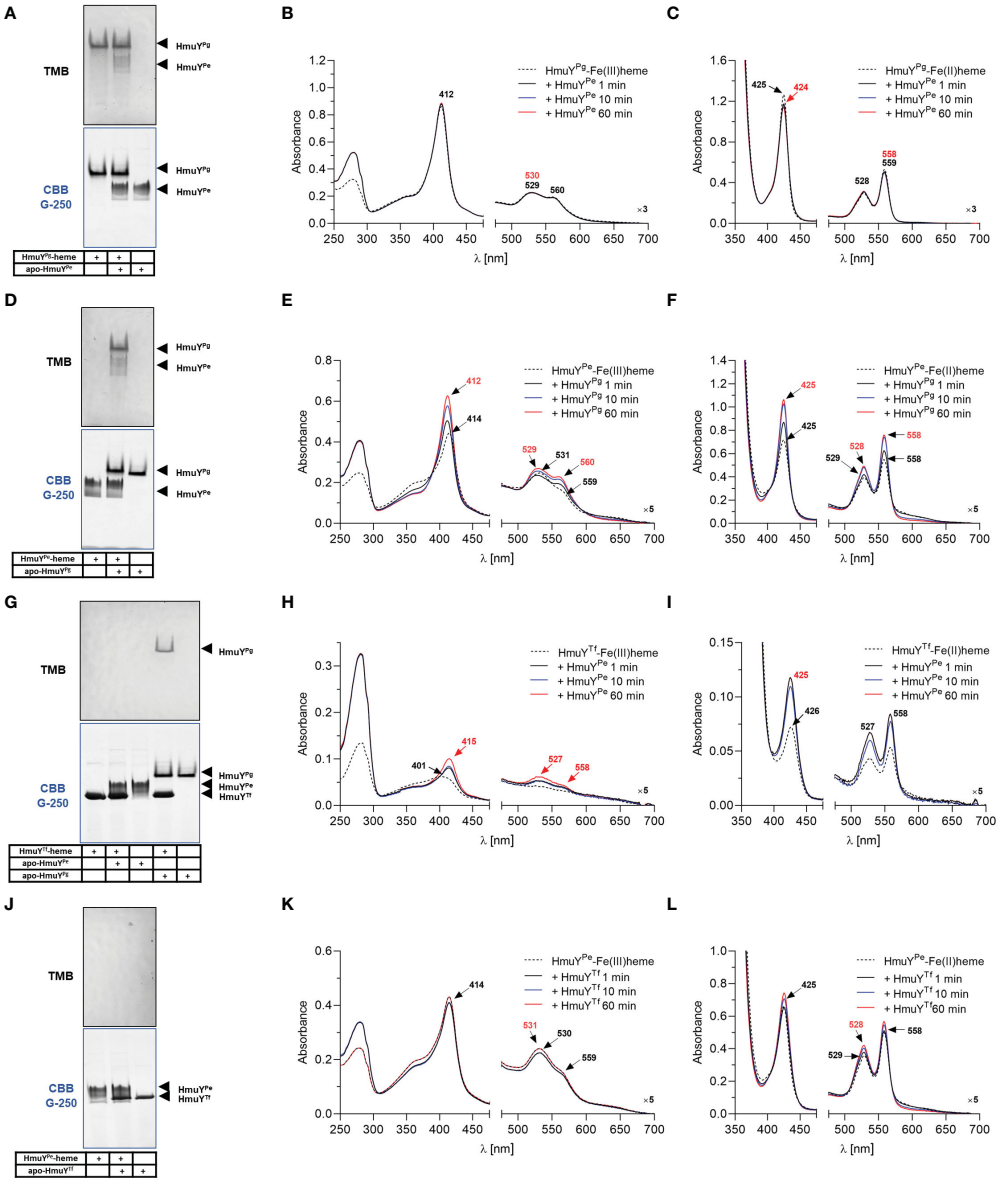
Heme sequestration capacity of *P. endodontalis* HmuY^Pe^ and HmuY homologs from *P. gingivalis* [HmuY^Pg^; **(A–F)**] or *T. forsythia* [HmuY^Tf^; **(G–L)**]. Heme transfer was examined using PAGE, staining with TMB-H_2_O_2_, and subsequent visualization of proteins by CBB G-250 staining **(A, D, G, J)**. *P. gingivalis* HmuY^Pg^ was used as a control. The HmuY^Pe^-heme complex after TMB-H_2_O_2_ staining is hardly visible due to analysis performed under aerobic conditions, causing lower heme binding to the HmuY^Pe^ under oxidizing conditions. Apo-proteins or proteins in complex with heme were incubated at equimolar concentrations under oxidizing **(B, E, H, K)** or reducing conditions **(C, F, I, L)**, the latter formed by 10 mM sodium dithionite. Changes in spectra were monitored using UV-visible absorbance spectroscopy.

To compare the strength of heme binding to the HmuY^Pe^ with other HmuY proteins, we used a competitive analysis with HmuY^Pg^ and HmuY^Tf^. For this purpose, we employed UV-visible spectroscopy and native electrophoresis (PAGE) (**Figure 4**). HmuY^Pe^ protein was unable to sequester heme complexed with HmuY^Pg^ (**Figures 4A–C**). At the same time, HmuY^Pg^ was able to capture heme from the HmuY^Pe^-heme complex under both oxidizing and reducing conditions (**Figures 4D–F**). In the case of HmuY^Tf^, we observed efficient heme sequestration of heme from the HmuY^Tf^-heme complex by HmuY^Pe^ under oxidizing and reducing conditions, while HmuY^Tf^ was not able to capture heme from the HmuY^Pe^ complex (**Figures 4G–L**). In contrast to the spectroscopic method, we could visualize neither HmuY^Pe^-heme nor HmuY^Tf^-heme complexes with PAGE because they were hardly visible after TMB-H_2_O_2_ staining (**Figures 4A, D, G, J**). This effect could be caused by oxidizing conditions applied in this experiment, resulting in weak heme binding or heme release.

### 3.4 Heme sources for *P. endodontalis*

**Figure 5:**
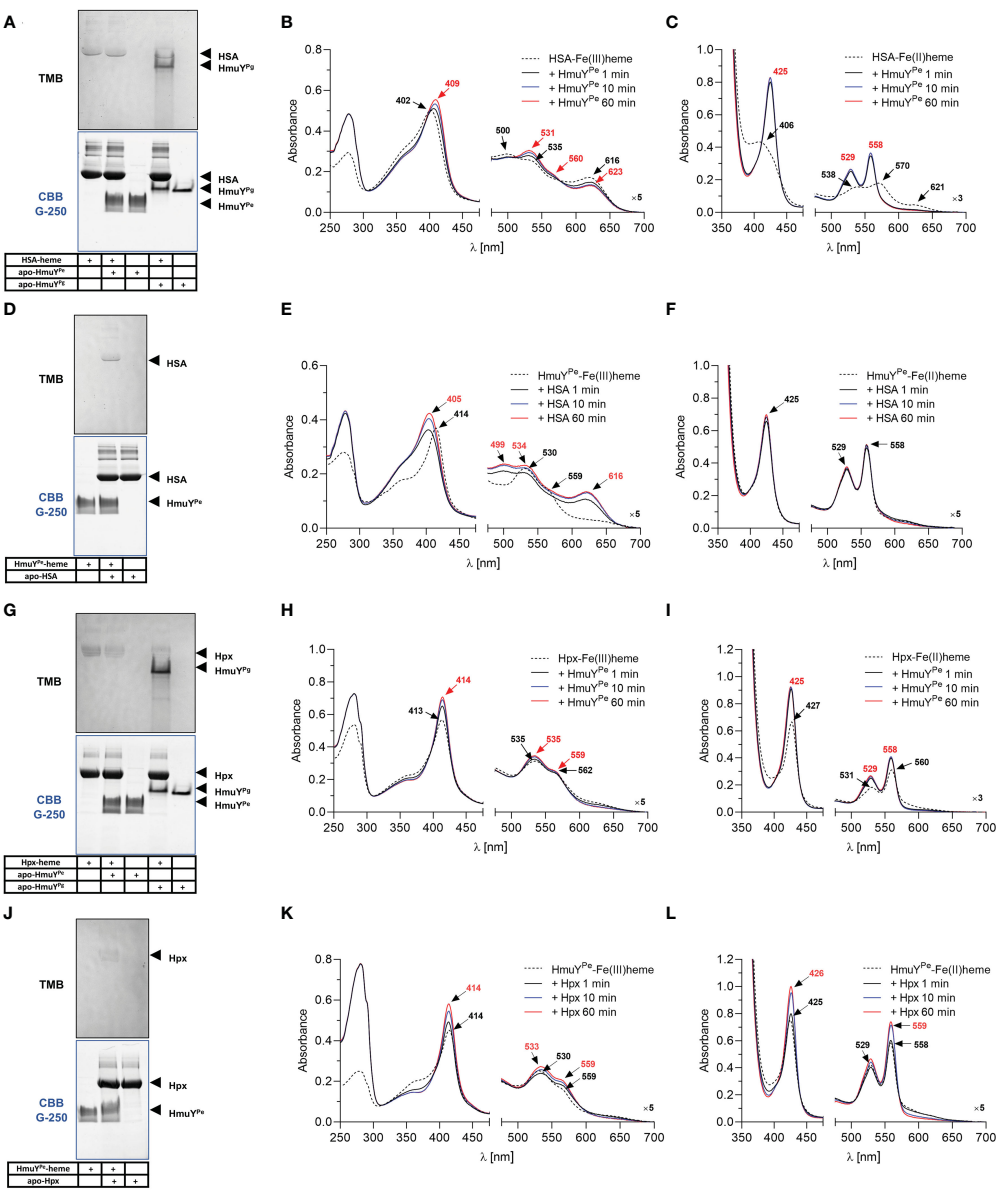
Heme sequestration capacity of *P. endodontalis* HmuY^Pe^ from human serum albumin [HSA; **(A–F)**] or hemopexin [Hpx; **(G–L)**]. Heme transfer was examined using PAGE, staining with TMB-H_2_O_2_, and subsequent visualization of proteins by CBB G-250 staining **(A, D, G, J)**. *P. gingivalis* HmuY^Pg^ was used as a control. The HmuY^Pe^-heme complex after TMB-H_2_O_2_ staining is hardly visible due to analysis performed under aerobic conditions, causing lower heme binding to the HmuY^Pe^ under oxidizing conditions. Apo-proteins or proteins in complex with heme were incubated at equimolar concentrations under oxidizing **(B, E, H, K)** or reducing conditions **(C, F, I, L)**, the latter formed by 10 mM sodium dithionite. Changes in spectra were monitored using UV-visible absorbance spectroscopy.

Further, we analyzed the ability of HmuY^Pe^ to directly sequester heme from host hemoproteins, which serve *in vivo* as the main heme source. Under oxidizing conditions, the affinity of HSA and HmuY^Pe^ to heme was similar because while using HSA-heme or HmuY^Pe^-heme complex with apo-form of the counterpart, we observed an equilibrium in heme binding between the proteins (**Figures 5A–F**). Although PAGE results are not clear (**Figures 5A, D**), using the spectroscopic method we observed the spectrum shift in two experimental settings (**Figures 5B, E**). Under reducing conditions, HmuY^Pe^ was able to capture heme bound to HSA, whereas apo-HSA could not sequester heme from HmuY^Pe^ (**Figures 5C, F**). In the case of Hpx, the results are inconclusive due to the low quality of HmuY^Pe^ TMB-H_2_O_2_ staining (**Figures 5G, J**). In the case of these proteins, equilibrium in Hpx-heme and HmuY^Pe^-heme complexes could also occur, which was confirmed by changes in the intensity of the analyzed UV-visible absorbance spectra over time (**Figures 5H, I, K, L**). However, since the absorbance maxima of both proteins in complex with heme are similar, we cannot draw firm conclusions. In contrast to HmuY^Pg^ (Smalley et al., 2011; Byrne et al., 2013; Śmiga et al., 2021), HmuY^Pe^ was unable to capture the heme associated with metHb (**Supplementary Figures S4A**, **S4B**). Moreover, heme sequestration was not observed after the chemical reduction of metHb (**Supplementary Figure S4C**). As a control, we used HmuY^Pg^, which can directly sequester heme complexed with HSA (**Figure 5A**; **Supplementary Figure S5**) (Smalley et al., 2011; Sieminska et al., 2021), Hpx (**Figure 5G**) (Bielecki et al., 2018), and metHb (**Supplementary Figure S4A**) (Smalley et al., 2011).

**Figure 6:**
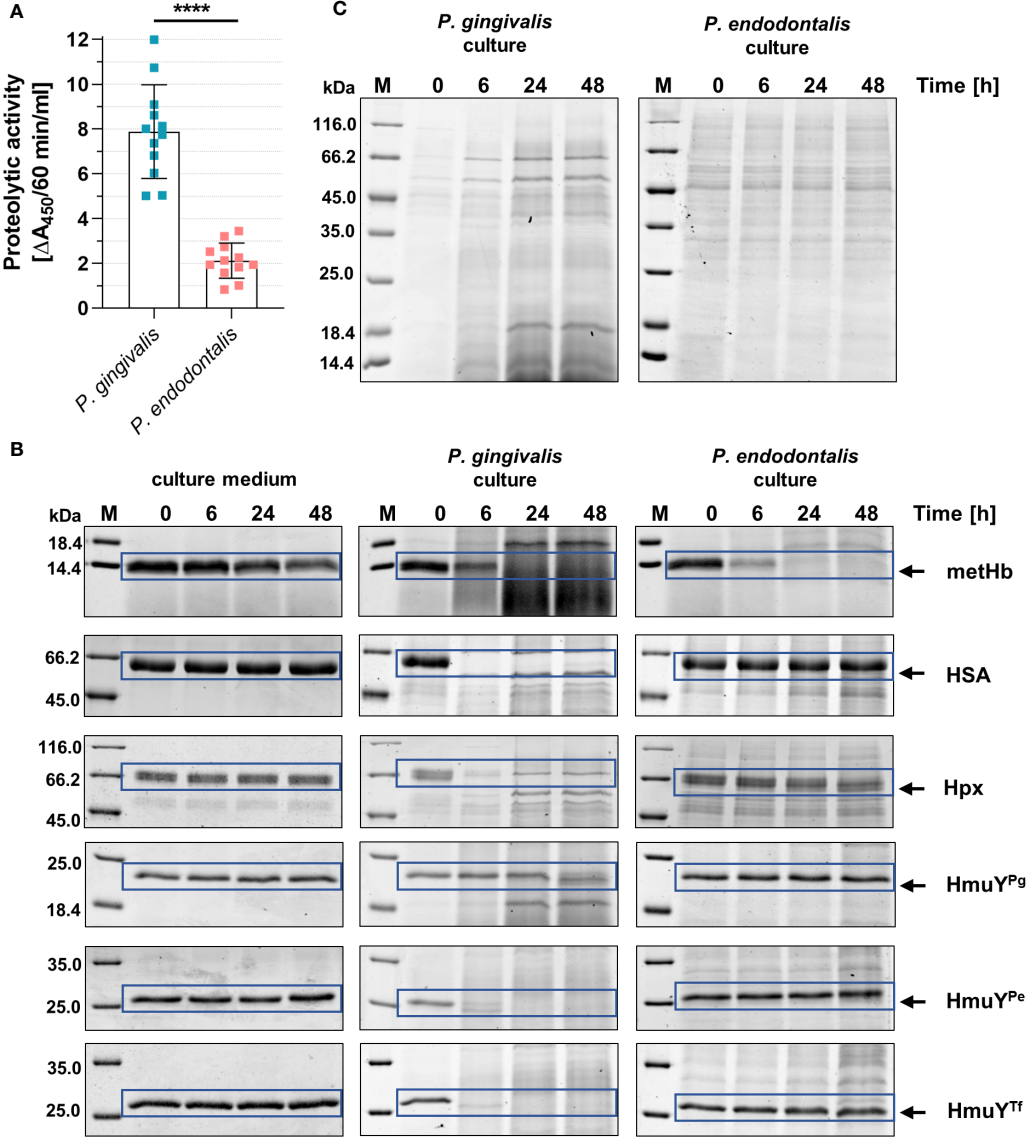
Comparison of the proteolytic activity of *P. endodontalis* and *P. gingivalis*. **(A)** Total proteolytic activity in whole bacterial cultures (iron- and heme-rich medium; Hm medium) was determined using azocasein as a substrate. The activity is shown as an increase in absorbance at 450 nm (A_450_) caused by the release of a colored product within 60 minutes by 1 ml of bacterial culture with an optical density at 600 nm of 1. **(B)** Susceptibility of human hemoproteins and selected HmuY proteins to degradation by proteases produced by *P. endodontalis* or *P. gingivalis*. Purified proteins were added to the bacterial cultures (Hm medium) at a final 2 µM concentration, samples were incubated, collected at the indicated time points, and analyzed by SDS-PAGE and CBB G-250 staining. As a control, Hm medium alone was used instead of bacterial cultures. **(C)** Protein pattern of *P. gingivalis* or *P. endodontalis* proteins in iron- and heme-rich culture medium (Hm medium) without adding host hemoproteins or HmuY proteins. metHb, methemoglobin; HSA, serum albumin; Hpx, hemopexin; HmuY^Pg^, *P. gingivalis* HmuY; HmuY^Pe^, *P. endodontalis* HmuY homolog; HmuY^Tf^, *T. forsythia* HmuY homolog; M, protein molecular mass markers. ****P<0.0001.

*P. gingivalis* can use a variety of heme sources thanks to the direct sequestration of heme from hemoproteins by HmuY^Pg^ or its synergistic cooperation with highly active proteases – gingipains (Smalley and Olczak, 2017; Śmiga et al., 2023b; Olczak et al., 2024). Although *P. endodontalis* does not encode gingipain homologs, it produces proteases, albeit less active compared to *P. gingivalis* proteases (**Figure 6A**). Nevertheless, they can efficiently degrade metHb in the *P. endodontalis* culture (**Figure 6B**). None of the additionally analyzed proteins were degraded by *P. endodontalis* proteases, including human hemoproteins and representatives of HmuY homologs produced by other human pathogens (**Figure 6B**). These results are consistent with *P. endodontalis* growing in the planktonic form with different heme sources, the best achieved in the presence of free heme or metHb (**Supplementary Figure S6**; **Figure 6C**).

### 3.5 *P. endodontalis* expresses the *hmuY^Pe^* gene under iron and heme starvation

**Figure 7:**
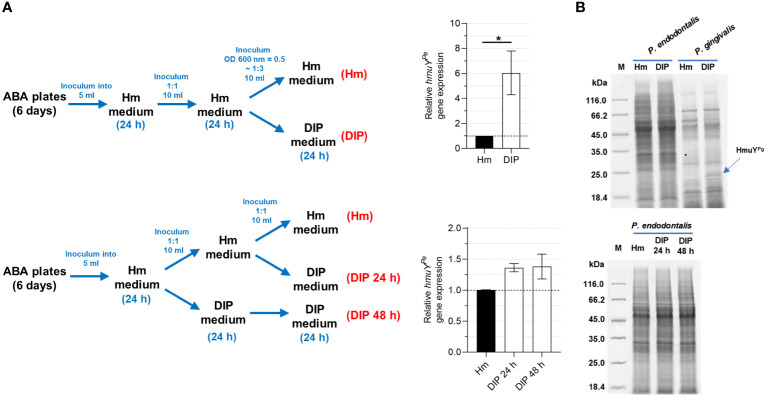
Influence of iron and heme starvation on *P. endodontalis* HmuY^Pe^ expression. **(A)** Determination of relative *hmuY^Pe^* gene expression examined by RT-qPCR with *16S rRNA* as a reference gene. Relative expression fold change represents mRNA levels in bacteria starved of iron and heme (DIP medium) *versus* bacteria grown in rich iron and heme conditions (Hm medium), the latter set as 1.0. **(B)** The influence of iron and heme starvation on the overall production of proteins in *P. endodontalis* was examined by SDS-PAGE and CBB G-250 staining. *P. gingivalis* was used as a control and a band indicating HmuY^Pg^ is shown with a blue arrow. M, protein molecular mass markers. *P<0.05.

Similar to other HmuY proteins that were characterized previously (Bielecki et al., 2018, 2020; Sieminska et al., 2021; Antonyuk et al., 2023), transcript encoding HmuY^Pe^ was produced at higher levels when bacteria were grown in iron- and heme-depleted conditions, formed as shown in **Figure 7A**. However, the increase of the *hmuY^Pe^* gene expression was only up to 6 times, whereas for *hmuY^Pg^* the expression increased up to several hundred times (Bielecki et al., 2018, 2020), resulting in a significant increase in produced HmuY^Pg^ protein (**Figure 7B**) (Olczak et al., 2010). This dissimilarity may result from weaker growth of *P. endodontalis* under laboratory conditions, especially under iron and heme starvation (**Supplementary Figure S6**).

### 3.6 HmuY proteins may serve as biological markers

Finally, we aimed to find whether, despite 50% amino acid sequence identity (**Figure 1**) and almost identical tertiary structures of HmuY^Pe^ and HmuY^Pg^ (**Figure 3C**), HmuY^Pe^ could potentially serve as a specific marker for *P. endodontalis*. We analyzed the purified HmuY^Pe^ protein, both in the form of native and denatured protein and showed cross-reactivity neither with IgG antibodies raised against HmuY^Pg^ nor HmuY^Tf^ (**Supplementary Figure S7**).

## 4 Discussion

Interactions between microorganisms and the host play an important role in the etiopathogenesis of many human diseases. Although the main ecological niches of *P. gingivalis* and *P. endodontalis* (subgingival pockets and root canals, respectively) are different, both species can be found in the same polymicrobial consortia, including periodontal pockets, often with other members of the Bacteroidota phylum (Kumar et al., 2003; Pereira et al., 2011; Lombardo Bedran et al., 2012; Lourenco et al., 2014). Proteomic analysis revealed that the type and number of proteins associated with virulence were more similar between *P. endodontalis* and the more virulent *P. gingivalis* W83 strain as compared to the less virulent *P. gingivalis* ATCC 33277 strain (Li et al., 2016). One of the main virulence factors expressed by the members of the Bacteroidota phylum is the Hmu heme acquisition system. Recently, we demonstrated that the expression of heme acquisition systems, mainly the Hmu system, is different between more and less virulent *P. gingivalis* strains, expressed and used more effectively in more virulent strains (Śmiga et al., 2024). Since one of its components, namely the HmuY protein is considered to be one of the main virulence factors of opportunistic pathogens, in this study, we performed a comparative analysis of the *P. endodontalis* HmuY^Pe^ protein and HmuY proteins produced by *P. gingivalis* (HmuY^Pg^) and *T. forsythia* (HmuY^Tf^).

The main heme sources for *P. endodontalis*, similar to *P. gingivalis*, are serum heme-sequestering proteins, heme complexed to bacterial proteins, and periodically, hemoglobin released from erythrocytes (Grenier and Mayrand, 1986; Sundqvist, 1992; Zerr et al., 1998, 2000, 2001; Smalley and Olczak, 2017). In humans, oral pathogens are provided with heme mainly by HSA, the main component of the gingival crevicular fluid. This is also the case of HmuY^Pe^ which captures heme directly from the HSA-heme complex, with higher ability under reducing conditions. This finding is consistent with literature data reporting *K_d_* of HSA-heme of 10^-7^-10^-8^ M, which is similar to that determined for the HmuY^Pe^, as well as by the fact that heme can be more easily released from the HSA-heme complex under reducing conditions (Cao et al., 2012). This property is also exploited by HmuY homologs in which two methionines are used to coordinate heme-iron (Bielecki et al., 2018, 2020; Sieminska et al., 2021; Antonyuk et al., 2023).

Although hemoglobin is unavailable in higher concentrations in the main niche occupied by *P. endodontalis*, namely root canals, this heme source can be available for this bacterium during treatment procedures, or when the bacterium is found in deep periodontitis sites, where in advanced stages of disease bleeding may occur. As shown in this study, even though the total proteolytic activity of *P. endodontalis* cultures was significantly lower compared to *P. gingivalis* cultures, the degradation capacity of metHb was comparable in both bacterial cultures. In contrast to *P. gingivalis*, which prefers to convert oxyhemoglobin possessing Fe(II)heme to metHb possessing Fe(III)heme, the process facilitating hemoglobin degradation by gingipains (Smalley et al., 2007, 2008) and direct heme sequestration by *P. gingivalis* HmuY^Pg^ (Smalley et al., 2011), HmuY^Pe^ was unable to sequester heme bound to metHb. It has been demonstrated that *P. endodontalis* can reduce metHb under anaerobic conditions (Zerr et al., 2001). However, we showed that this ability does not allow HmuY^Pe^ for heme sequestration from chemically reduced metHb in the experiment carried out under limited oxygen access. Therefore, based on better *P. endodontalis* growth in a culture medium supplemented with metHb, we assume that the high ability of metHb degradation by this bacterium may be used to efficiently release heme from metHb which can be subsequently bound by HmuY^Pe^, instead of direct heme capture by HmuY^Pe^.

*P. gingivalis* expresses several features engaged in its higher adaptation to the changing host environment and inhibitory activity against other bacteria, including *P. endodontalis* (van Winkelhoff et al., 1987). Many *Porphyromonas* species are obligate anaerobes and can survive temporarily in the presence of oxygen, *P. gingivalis* being the best example (Smalley et al., 1998; Mark Welch et al., 2016; Slezak et al., 2020). But this is not the case for *P. endodontalis* which is highly sensitive to oxygen (van Winkelhoff et al., 1985b, 1986). Data obtained in our study showed that one of the *P. gingivalis* adaptive features can be assigned not only to better tolerance to oxygen and higher proteolytic activity but also to different properties of heme binding by HmuY^Pg^ as compared to HmuY^Pe^. The strength of heme binding by HmuY proteins depends on the redox state of the external environment (Olczak et al., 2024) and based on our results is as follows: HmuY^Pg^>HmuY^Pe^>HmuY^Tf^. HmuY^Pg^, coordinating heme iron with two histidines, binds heme efficiently under both oxidizing and reducing conditions. When two methionine residues are involved in this process in HmuY homologs, including *T. forsythia* HmuY^Tf^, they bind heme preferentially under reducing conditions (Olczak et al., 2024). *P. endodontalis* HmuY^Pe^, coordinating heme-iron with the histidine-methionine pair, is a missing link between the HmuY proteins mentioned above. Although *P. endodontalis* HmuY^Pe^ is the first characterized protein among HmuY family members with a histidine-methionine pair engaged in heme-iron coordination, our theoretical analysis showed that histidine-methionine pair may be commonly used by HmuY homologs from other *Porphyromonas* species. This feature allows HmuY^Pe^ and possibly other HmuY homologs for better heme sequestration from other hemophore-like proteins that use two methionines to coordinate heme-iron, for example from *T. forsythia* HmuY^Tf^, allowing *P. endodontalis* and other *Porphyromonas* species to compete for heme source. However, HmuY^Pe^ is unable to capture heme bound to HmuY^Pg^, which confirms the predominance of *P. gingivalis* in heme acquisition in polymicrobial consortia.

Identification and characterization of bacterial antigens expressed by the members of the Bacteroidota phylum allows not only for improved knowledge of pathogens’ phenotypes and their pathogenicity but also for the development of diagnostic and therapeutic strategies. One of the targets in such methods can be the host’s immune response toward components of bacterial heme acquisition systems, including HmuY proteins. The amino acid residues that form the core of the protein structure in HmuY family members are most conservatively preserved, whereas the greatest variability of epitopes exists on the surface of HmuY proteins (Śmiga et al., 2023a). Previously, in patients with periodontitis, we reported higher serum levels of antibodies directed against total *P. gingivalis* antigens and HmuY^Pg^ protein, as well as HmuY homolog from *P. intermedia* (HmuY^Pi-2^) (Trindade et al., 2012; Śmiga et al., 2023a). Others demonstrated that in patients with apical periodontitis, *P. gingivalis* and *P. endodontalis* correlated with higher serum levels of IgG antibodies directed toward both bacterial species (Flynn et al., 2012). Importantly, we showed that there is no cross-reactivity between *P. gingivalis* HmuY^Pg^ and *T. forsythia* HmuY^Tf^ (Śmiga et al., 2015; Śmiga et al., 2023a) and that IgG antibodies raised toward these proteins did not recognize HmuY^Pe^ (this study). This suggests that HmuY proteins produced by oral pathogens differ in their epitopes sufficiently to be considered candidates for the development of diagnostic methods or biological markers to monitor polymicrobial diseases.

## Data availability statement

The raw data supporting the conclusions of this article will be made available by the authors, without undue reservation.

## Author contributions

MŚ Conceptualization, Data curation, Formal Analysis, Investigation, Methodology, Project administration, Supervision, Validation, Visualization, Writing – original draft, Writing – review & editing. TO: Conceptualization, Formal Analysis, Funding acquisition, Validation, Writing – original draft, Writing – review & editing.
