# Supplementary material for: Porphyromonas endodontalis HmuY differentially participates in heme acquisition compared to the Porphyromonas gingivalis and Tannerella forsythia hemophore-like proteins
Source: Front Cell Infect Microbiol. 2024 Jun 13;14:1421018. doi: 10.3389/fcimb.2024.1421018 (PMC11208336; doi:10.3389/fcimb.2024.1421018)
Supplement: Supplementary file 1 [file DataSheet_1.pdf]

Supplementary material

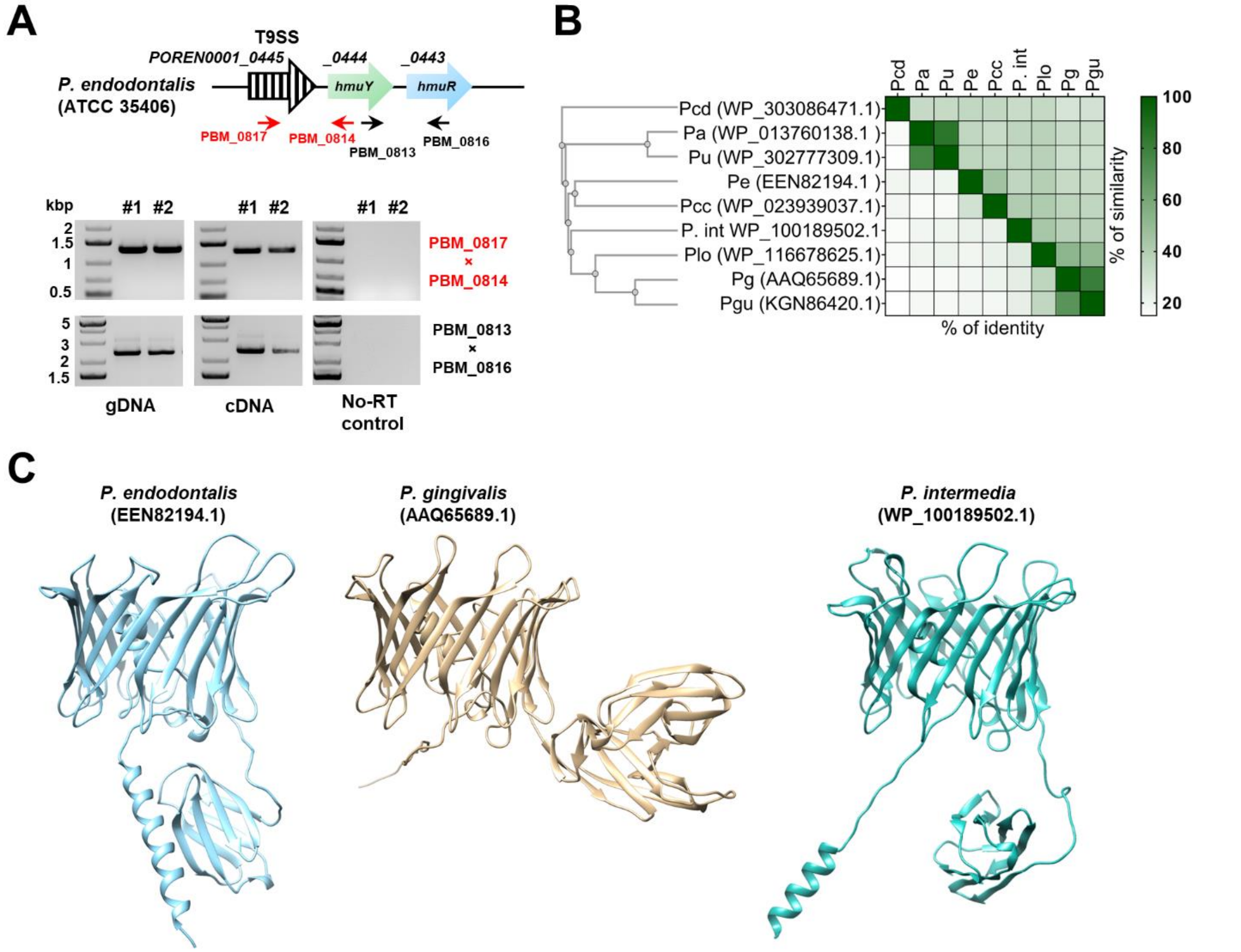

Figure S1. Characterization of *P. endodontalis POREN0001_0445* gene. (A) Transcript analysis was performed with PCR and primers amplifying fragments of *POREN0001_0445* and *hmuY*^{Pe} (PBM_0817 and PBM_0814) region or *hmuY*^{Pe} and *hmuR*^{Pe} (PBM_0813 and PBM_0816) region using genomic DNA (gDNA) and cDNA with No-RT control. (B) Amino acid sequences of the protein encoded by the *POREN0001_0445* gene and homologous proteins identified in selected *Porphyromonas* species (Pcd – *P. circumdentaria*, Pa – *P. asaccharolytica*, Pu – *P. uenonis*, Pe – P. *endodontalis*, Pcc – *P. crevioricanis*, Plo – *P. loveana*, Pg – *P. gingivalis*, Pgu – *P. gulae*) and *P. intermedia* (*P. int*) were used for phylogenetic analysis. The identity and similarity between proteins (%) are shown as a heat map. The simplified guide tree was created with Clustal Omega (https://www.ebi.ac.uk/jdispatcher/msa/clustalo). (C) Comparison of the predicted structure models of *P. endodontalis* POREN0001_0445 protein with homologs identified in *P. gingivalis* and *P. intermedia*.

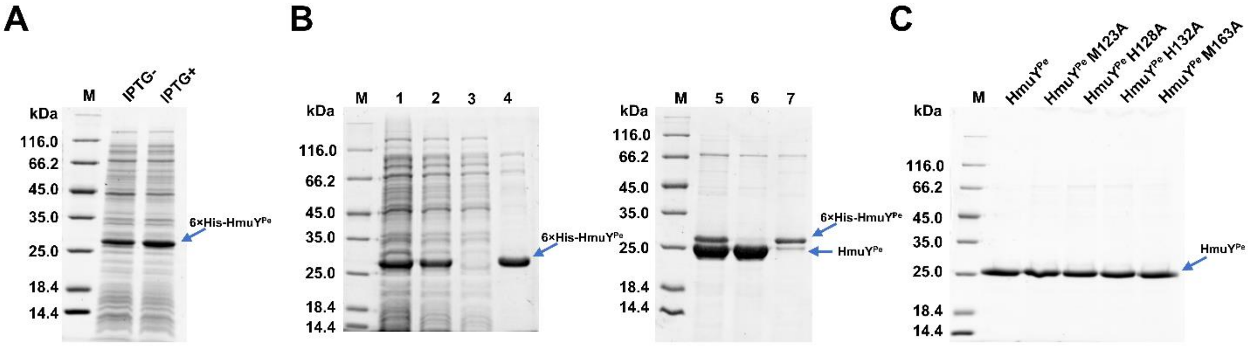

Figure S2. Overexpression and purification of *P. endodontalis* $HmuY^{Pe}$ protein and its site-directed mutagenesis variants. (A) $HmuY^{Pe}$ tagged with 6 histidines at the N terminus (6×His-$HmuY^{Pe}$) was overexpressed in *E. coli* after induction with IPTG and the bacterial cultures were collected before (IPTG-) and after (IPTG+) induction. (B) $HmuY^{Pe}$ was purified using cobalt-immobilized resin (TALON Superflow). Bacterial lysates (1) were centrifuged, 6×His-$HmuY^{Pe}$ protein was purified from the soluble fraction (2), and the efficiency of protein binding to the resin was verified by analyzing the unbound protein fraction (3). 6×His-$HmuY^{Pe}$ protein was eluted from the resin using imidazole (4). To cleave off the 6×His tag, factor Xa was used (5). The protein was then incubated with nickel-immobilized resin (Ni-NTA), separating the purified $HmuY^{Pe}$ (6) from 6×His-$HmuY^{Pe}$ (7). (C) Site-directed mutagenesis variants of the $HmuY^{Pe}$ protein (M123A, H128A, H132A, and M163A) were overexpressed and purified using the same procedures. Proteins were separated by SDS-PAGE and stained with CBB G-250.

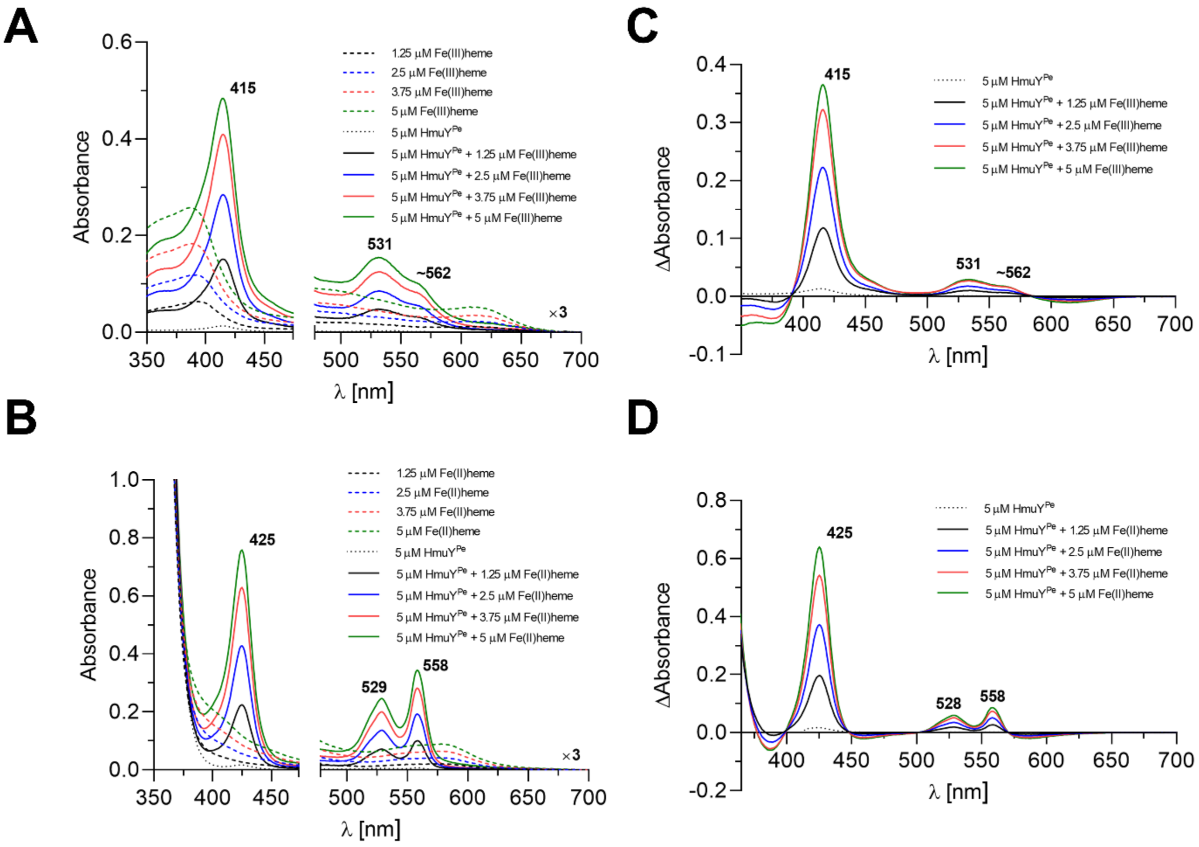

Figure S3. Titration of the HmuY^{Pe} protein with heme. 5 µM protein was titrated with increasing concentration of heme under oxidizing (A) and reducing (B) conditions (the latter obtained by the addition of 10 mM sodium dithionite). Complex formation was monitored by UV-visible absorbance spectroscopy. The difference absorbance spectra were analyzed under oxidizing (C) and reducing (D) conditions.

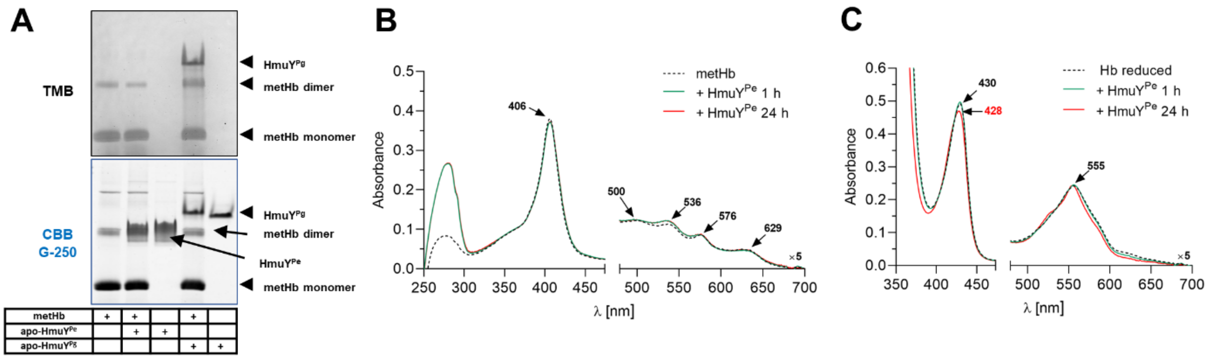

Figure S4. Heme sequestration capacity of *P. endodontalis* $HmuY^{Pe}$ from hemoglobin. Heme transfer from methemoglobin (metHb) or reduced metHb (Hb red) was examined using PAGE, staining with TMB-H_2O_2, and subsequent visualization of proteins by CBB G-250 staining (A). *P. gingivalis* $HmuY^{Pg}$ was used as a control. Hemoglobin and $HmuY^{Pe}$ were incubated at equimolar concentrations under oxidizing (B) and reducing conditions (C), the latter formed by 10 mM sodium dithionite. Spectra were monitored using UV-visible absorbance spectroscopy.

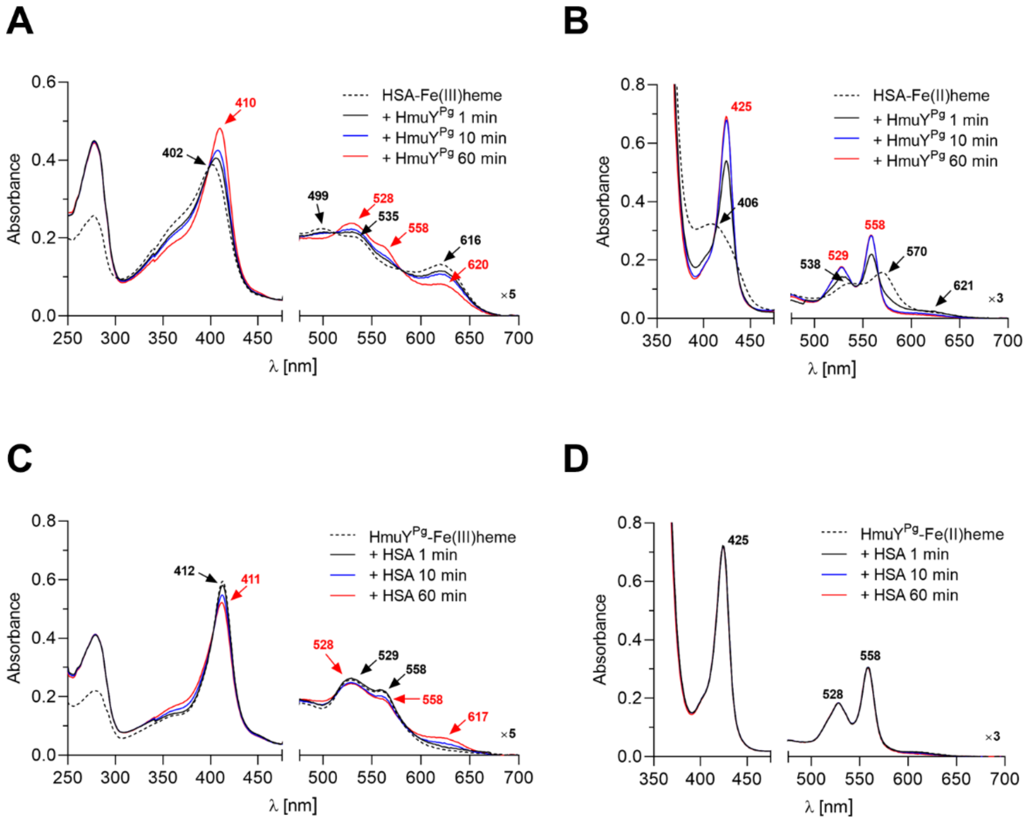

Figure S5. Heme sequestration capacity of *P. gingivalis* HmuY^{Pg} from human serum albumin (HSA). Heme transfer from HSA was examined using UV-visible absorbance spectroscopy. HSA and HmuY^{Pg} were incubated at equimolar concentrations under oxidizing (A, C) and reducing conditions (B, D), the latter formed by 10 mM sodium dithionite.

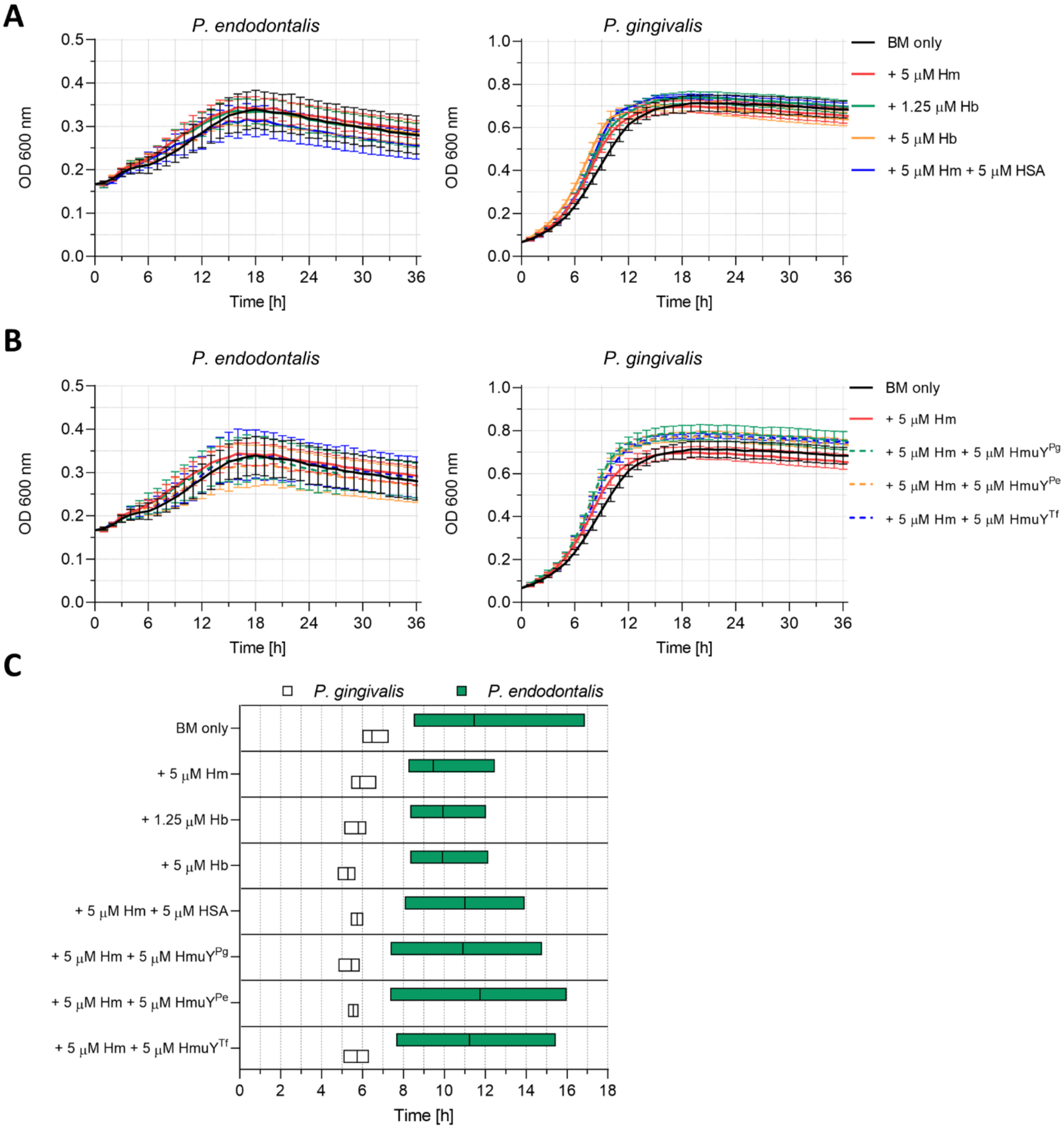

Figure S6. *P. endodontalis* and *P. gingivalis* growth in culture media supplemented with various heme sources. Bacteria were cultured for two passages in a basal medium (BM) without adding a heme source (BM only). Then, bacteria were cultured in BM with the addition of heme, host hemoproteins (A, C), or selected HmuY proteins (B, C). Bacterial growth was monitored by measuring the optical density at 600 nm (OD 600 nm) over time and the results are shown as mean ± standard error. (C) Comparison of the bacterial growth rate depending on the heme source, determined by measuring the time required to reach an optical density at 600 nm of 0.25 by bacterial cultures, indicating the onset of the mid-log growth phase. Results are shown as the mean measured time (h) and the time range (min and max values). Hm, heme; Hb, human hemoglobin; HSA, human serum albumin; $HmuY^{Pg}$, *P. gingivalis* HmuY; $HmuY^{Pe}$, HmuY homolog from *P. endodontalis*; $HmuY^{Tf}$, HmuY homolog from *T. forsythia*.

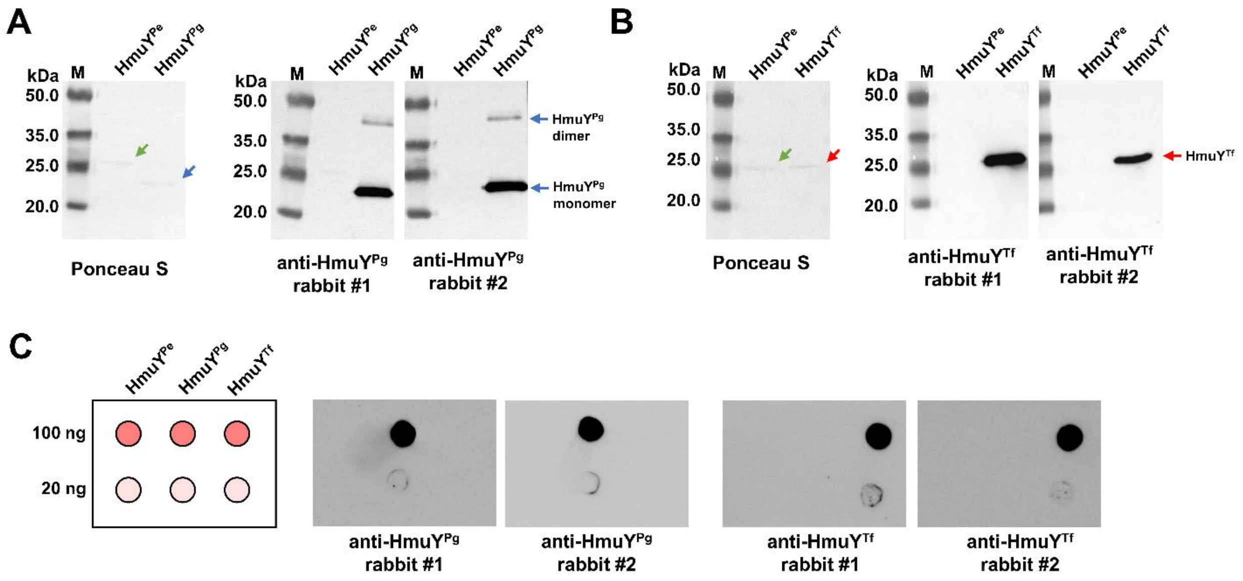

Figure S7. Analysis of immunogenic epitopes and their cross-reactivity in denatured and native proteins. The reactivity of purified *P. endodontalis* HmuY^{Pe} with anti-HmuY^{Pg} (A) or anti-HmuY^{Tf} (B) antibodies was examined using Western blotting and chemiluminescence staining. Proteins (100 ng) were separated by SDS-PAGE, transferred onto a nitrocellulose membrane, and visualized with Ponceau S staining. Purified HmuY^{Pg} or HmuY^{Tf} protein was examined as a control. HmuY^{Pe}, HmuY^{Pg}, and HmuY^{Tf} are indicated with green, blue, and red arrows, respectively. Dot blotting analysis was performed using 20 and 100 ng of proteins (C). Antibodies from two immunized rabbits were used (#1 and #2). M, protein molecular mass marker.
