# Supplementary material for: Porphyromonas endodontalis HmuY differentially participates in heme acquisition compared to the Porphyromonas gingivalis and Tannerella forsythia hemophore-like proteins
Source: Front Cell Infect Microbiol. 2024 Jun 13;14:1421018. doi: 10.3389/fcimb.2024.1421018 (PMC11208336; doi:10.3389/fcimb.2024.1421018)
Supplement: Supplementary file 2 [file DataSheet_2.pdf]

Supplementary material

Table S1. Primers designed and used in this study.

**Primer name**	**DNA sequence 5'→3'**	**Gene abbreviation (locus ID)**	**Description**
PBM_0801	ACACCATCACCACCATCACTTAATCGAGG GAAGGAACCAAACTCCTAAACCCGG	*hmuY*^{Pe} (*POREN0001_0444*)	amplify *P. endodontalis hmuY*^{Pe} gene sequence without the predicted signal peptide, used to clone into XmnI and BamHI restriction sites of pTriEx-4 plasmid
PBM_0802	GAGATCTGAGAATTCGGATCCTTACTTCA CACTCACATTATAGTCGTA		
PBM_0803	CTATAACCATTAAGTTTAGCGCGGGAGGT GGCTCTCACGATA	*hmuY*^{Pe} (*POREN0001_0444*)	used to generate pTriEx-4 plasmid encoding *P. endodontalis* HmuY^{Pe} protein with methionine 123 (M123) replaced with alanine
PBM_0804	ACTATCGTGAGAGCCACCTCCCGCGCTAA ACTTAATGGTTAT		
PBM_0805	ATGGGAGGTGGCTCTGCCGATAGTAATCA CGAGCAAACAGG	*hmuY*^{Pe} (*POREN0001_0444*)	used to generate pTriEx-4 plasmid encoding *P. endodontalis* HmuY^{Pe} protein with histidine 128 (H128) replaced with alanine
PBM_0806	TGTTTGCTCGTGATTACTATCGGCAGAGCC ACCTCCCATGC		
PBM_0807	TGGCTCTCACGATAGTAATGCCGAGCAAA CAGGTTACAATCATC	*hmuY*^{Pe} (*POREN0001_0444*)	used to generate pTriEx-4 plasmid encoding *P. endodontalis* HmuY^{Pe} protein with histidine 132 (H132) replaced with alanine
PBM_0808	TGATTGTAACCTGTTTGCTCGGCATTACTA TCGTGAGAGCCACC		
PBM_0809	TGATTACGACCTTGGCAACGCGCCTCCCC GCGTTCGTTTGTC	*hmuY*^{Pe} (*POREN0001_0444*)	used to generate pTriEx-4 plasmid encoding *P. endodontalis* HmuY^{Pe} protein with methionine 163 (M163) replaced with alanine
PBM_0810	AACGAACGCGGGGAGGCGCGTTGCCAAG GTCGTAATCAAGCC		
PBM_0811	CGTGAGGTGTCGGCTTAAGT	*16S rRNA* (*NR_042803*)	amplify a fragment of the *P. endodontalis 16S rRNA* gene in RT-qPCR analysis
PBM_0812	CTTCCCTTTGTCCCTGCCAT		
PBM_0813	CCAACTCCTAATCCTCCCGC	*hmuY*^{Pe} (*POREN0001_0444*)	amplify a fragment of the *P. endodontalis hmuY*^{Pe} gene in RT-qPCR analysis
PBM_0814	TTCGCCACAATTGAGACGGA		
PBM_0816	CTAGCAAGGCAGAGTGTGGG	*hmuR*^{Pe} (*POREN0001_0443*)	used in PCR to confirm gene co-transcription
PBM_0817	CGTCCTAGGATGGCTACAGG	*POREN0001_0445*	
